# The facts about the effects of pedagogical agents on learners’ cognitive load: a meta-analysis based on 24 studies

**DOI:** 10.3389/fpsyg.2025.1635465

**Published:** 2025-07-24

**Authors:** Hao Li, Zhuo Wang, Long Ding, Jiwu Zhang, Guohua Wang

**Affiliations:** ^1^School of Teacher Education, Qiqihar University, Qiqihar, China; ^2^Faculty of Education, School of Educational Technology, Henan Normal University, Xinxiang, China

**Keywords:** multimedia learning, cognitive load, instructional design, pedagogical agent, meta-analysis

## Abstract

The role of pedagogical agents in multimedia learning as an element that gives students social cues to promote cognition has been demonstrated in terms of enhancing students’ academic performance and intrinsic motivation. Cognitive load is an important factor in measuring the effectiveness of multimedia learning, but the results of pedagogical agents’ effects on learners’ cognitive load have been consistently inconsistent. In this study, a meta-analytical approach was used to comprehensively analyse 24 empirical studies to investigate the effects of pedagogical agents on students’ cognitive load in multimedia learning and the moderating factors in their process. The results showed that the teaching agent only slightly reduced the cognitive load. Moderating analyses show that the pedagogical agent appearance, pedagogical agent role, subject domain, form of media mix, intervention duration, and learning pace play a moderating role in the process of the pedagogical agent’s influence on cognitive load, and can significantly reduce the cognitive load of learners.

## 1 Introduction

In recent years, with the development of image processing technology and virtual reality technology, pedagogical agents have been widely introduced into multimedia learning environments (Li et al., 2023). Pedagogical Agent is a real person or virtual character that performs a pedagogical role in digital learning environments, which often communicates with learners as a teacher or a peer using language or mental emotions, with the aim of providing learners with a socially perceptive learning experience (Pi et al., 2023). Despite the proven effectiveness of pedagogical agents in enhancing students’ academic performance, motivation, and engagement, their effects on learners’ cognitive load have not yet yielded consistent results. Some researchers do not even support the use of instructional agents in multimedia learning, arguing that it may increase learners’ cognitive load, which in turn affects the reliability of results such as learning engagement, intrinsic motivation, etc. (Choi and Clark, 2006; Dinçer and Doğanay, 2017). In this study, we will use meta-analysis to integrate the results of national and international experimental studies related to measuring learners’ cognitive load with the presence of instructional agents as a dependent variable, and to analyse the main effect of instructional agents on students’ cognitive load and the moderators that may have an impact on the results.

## 2 Related work

### 2.1 An overview of the effects of pedagogical agents on cognitive load

Many scholars have carried out a lot of research on how pedagogical agents affect cognitive load, forming the following three views: pedagogical agents have a significant reduction of students’ cognitive load; pedagogical agents have a significant increase of students’ cognitive load; and pedagogical agents do not have a significant effect on students’ cognitive load. In this study, hypotheses were made based on each of these three perspectives.

*H1*: Pedagogical agents have a significant mitigating effect on students’ cognitive loads.

Embodiment theory suggests that pedagogical agents in online courses activate learners’ social perception of interacting with another social entity (Mayer, 2014a). According to the Cognitive-Emotional–Social theory social processes influence the selection of verbal information as well as the organization of information, and learners explicitly focus their attention on the learning content by comprehending the information conveyed by the pedagogical agent, which facilitates the efficient use of cognitive resources and promotes the processing of the learning material for memory. There is also a part of research that validates this idea, De Melo et al. (2020) used augmented reality-based embodied pedagogical agent to improve learning performance and reduce the cognitive load of learners. A study by Wang et al. (2020a) also showed that students perceived less mental effort when the pedagogical agent was on-screen and used a more intuitive EEG physiological measure to conclude that a pedagogical agent in a lecture video significantly reduced students’ cognitive load.

*H2*: Pedagogical agents have a significant increase in students’ cognitive load.

However, it has been argued that teaching agents may generate additional cognitive load or lead to distraction effects, both of which may be detrimental to learning outcomes. Also according to The Cognitive-Affective-Social Theory of Learning in digital Environments (CASTLE), the presence of an agentic image triggers social processes that affect the selection and organization of non-verbal information, which in turn affects working memory capacity during learning (Schneider et al., 2022a). Learners must not only process the learning content, but also cognitively process the pedagogical agent, paying for additional working memory resources. Mayer (2014b)also noted that too many social cues can overload the visual channel of working memory. As learners must divide their attention between the instructional agent and the instructional message, this can lead to a distraction effect that inhibits learning. Therefore, social cues can be viewed as an additional working memory load due to the design of multimedia learning materials, also known as irrelevant cognitive load. In a study by Lusk and Atkinson (2007), a learning system without a pedagogical agent allowed learners to perceive a lower cognitive load compared to a learning system with a pedagogical agent. There have also been studies using eye-tracking techniques to investigate the relationship between distraction and the visibility of pedagogical agents. By recording the eye movements of participants who watched instructional videos, the researchers found that the presence of the instructional agent distracted students from other visual elements in the video. The results of an eye-tracking study by Wang and Antonenko (2017) also showed that students’ visual attention was clearly distracted by pedagogical agents during a simple learning task.

*H3*: Pedagogical agent has no significant effect on students’ cognitive load.

The above two positions on how pedagogical agents affect students’ cognitive load are actually not contradictory. On the one hand, pedagogical agents give learners social perception facilitating cognitive processes; on the other hand, pedagogical agents as additional visual elements increase cognitive load. The result of the two effects working together is reflected in a considerable number of empirical studies, i.e., non-significant experimental results. The animated pedagogical agent system used by Yung and Paas (2015) to teach biology is junior high school did not significantly improve students’ cognitive load, although it improved academic performance. Schroeder et al. (2017) also did not find that pedagogical agents significantly reduced learners’ cognitive load or prevented distraction effects using an agent role measurement tool. There are many more studies like this that report insignificant effects of instructional agents on cognitive load, and it is even reasonable to assume that a subset of these studies have non-significant results due to unavoidable publication bias.

### 2.2 Moderating variables of instructional agents affecting cognitive load

As mentioned earlier, pedagogical agents have a mixed effect on students’ cognitive load, which may be due to the characteristics embodied in pedagogical agents, the inherent complexity of learning materials, and learner factors. Therefore the factors that play a moderating role in the process of pedagogical agents affecting learners’ cognitive load should be considered in depth, and therefore this study will examine the role of the following nine moderating factors based on previous research.

#### 2.2.1 Pedagogical agent appearance

The appearance of pedagogical agents usually includes three categories: cartoon, anthropomorphic, and real. These shapes may affect students’ affective experience and attention. For example, cartoon shaped agents may attract students’ attention through fun and thus reduce cognitive load, whereas anthropomorphic or real-life shapes may facilitate learning by enhancing the agent’s relatability and authenticity. In addition, the shape of the agent has a significant effect on students’ initial attraction and interest, but whether different shapes affect cognitive load remains controversial (Choi and Clark, 2006).

#### 2.2.2 Pedagogical agent roles

Pedagogical agents can be categorized into explanatory and guiding agents based on their role. Explanatory agents help students understand content by directly teaching knowledge and concepts, but excessive explanation may increase the cognitive load on the learner (Moreno et al., 2001). Guided agents, on the other hand, use strategies such as question guidance and prompting to encourage students to construct knowledge on their own, which may be more helpful in reducing cognitive load. Guided agents are believed to be able to help students think more deeply through provocative questions and timely prompts, but this may also increase the load on students by over-guiding.

#### 2.2.3 Pedagogical agent voices

Voices of pedagogical agents fall into two main categories: live-action voiceovers and machine synthesis. Research suggests that live voices may be more relatable than machine synthesised voices, increasing student engagement and emotional acceptance, thus reducing cognitive load to some extent. However, machine-synthesised voices, although consistent and controllable, may also lack emotional layers that make it difficult for students to remain focused for long periods of time, thus increasing cognitive load (Mayer, 2014a). Thus real-life voiceovers and machine-synthesized voices may affect cognitive load differently.

#### 2.2.4 Pedagogical agent expressions

Facial expressions of pedagogical agents can be categorized as either expressive or non-expressive. Expressive agents can help students understand the content by making the message more vivid through facial expression changes (Wang, 2022). However, too many or unnecessary expressions may lead to distraction and increase unnecessary cognitive load (Schneider et al., 2022b). Thus, the presence or absence of expressions in a teaching agent may have different effects on the cognitive load of the learner, depending on how appropriately facial expressions are used.

#### 2.2.5 Pedagogical agent body movements

An agent’s body movements, including gestures, instructions, and somatic expressions, are often thought to enhance the effectiveness of message delivery. Appropriate movement can enhance understanding of the content (Mayer and DaPra, 2012). However, too much body movement may lead to information interference and increase the cognitive load on students. Thus body movements may modulate the allocation of students’ attention to some extent and have an impact on the cognitive load of learners.

#### 2.2.6 Subject domain

The use of pedagogical agents may affect students’ cognitive load depending on the type of subject domain. For subject domains such as natural sciences and engineering and technology, which are mainly systematic and logical, pedagogical agents may help students to reduce their cognitive load through concrete demonstrations and step-by-step explanations; whereas in fields such as humanities and social sciences, where the abstractness and complexity of the content may require pedagogical agents to be more flexible in order to adapt to different types of knowledge transfer, the effectiveness of pedagogical agents may vary significantly across subject domains (Mayer, 2002).

#### 2.2.7 Form of media mix

In multimedia learning, the form of media combination used by the pedagogical agent may directly affect the learners’ information processing process. When content is presented as a combination of pictures and text, students may need to switch between visual and textual information, resulting in a higher cognitive load. In contrast, a single presentation form of pictures or text helps to reduce information interference and thus cognitive load, facilitating a smoother learning process. Therefore, differences in the form of media combination can significantly modulate learners’ cognitive processing load (van Merriënboer and Sweller, 2010).

#### 2.2.8 Intervention duration

Different intervention durations may have different effects on students’ cognitive load. Shorter intervention durations may help maintain learners’ attention but may lead to inadequate information processing, while longer intervention durations may provide more comprehensive learning opportunities but may increase cognitive fatigue (van Merriënboer and Sweller, 2005).

#### 2.2.9 Learning pace

Learning pacing can be categorized into system pacing and self-paced pacing. Systematic pacing, in which the system presets the pace and the student has no control over the learning process, may be appropriate for the transfer of knowledge with simpler content, but may lead to an increase in cognitive load. Self-paced learning, on the other hand, allows students to adjust the learning process according to their own pace, which helps to reduce the cognitive load and is particularly suitable for learning complex content (Koć-Januchta et al., 2020). The availability of self-pacing may be important for the effectiveness of cognitive load reduction for learners.

In summary, this study uses a meta-analytical approach in an attempt to address the following questions:

*RQ1*: What kind of influence does pedagogical agent have on learners’ cognitive load as a whole?

*RQ2*: Are there significant differences in the effects of pedagogical agents on cognitive load in terms of moderating variables such as agent appearance, role, voice, expression, body movement, subject domain, form of media mix, intervention time, and learning pace?

## 3 Materials and methods

### 3.1 Inclusion and exclusion criteria

This study’s compliance with cooper’s criteria established strict screening conditions as follows: (1) It must be an empirical study investigating learners’ cognitive load. (2) The independent variable of the study must be the presence or absence of an instructional agent, the dependent variable must be cognitive load, and the experiment must include at least two sets of controlled experiments (with and without an agent). (3) The study must provide sufficient statistical data to calculate the effect size, such as the mean, standard deviation, and sample size of each group (4) Only experimental results using authoritative cognitive load scales were considered, including the Paas Mental Effort Rating Scale (Paas et al., 1994; NASA-TLX, 2025), and Subjective Cognitive Load Scale (Leppink et al., 2013), will be considered to ensure reliability of results. (5) Publish in English.

### 3.2 Search strategy

The literature search was conducted in Web of Science, Elsevier Science Direct, and Springer with the search formula of the Boolean operator “TS = (cognitive load or cognitive workload or mental workload or mental effort or perceived difficulty or perceived ease) AND TS = (pedagogical agent or animated pedagogical agent or embodied pedagogical agent or virtual pedagogical agent or instructor) AND TS = (multimedia learning or instructional video or education video)” and matches were retrieved only in the title, abstract, and keywords. Search year ends 30 December 2024. Together with the 15 records we collected from other sources (e.g., books), a total of 544 bibliographic records were retrieved. Finally, we inquired about unpublished manuscripts from researchers in the field. We also contacted authors who did not provide specific data in their articles to obtain the original data, but did not receive a response.

**Figure 1:**
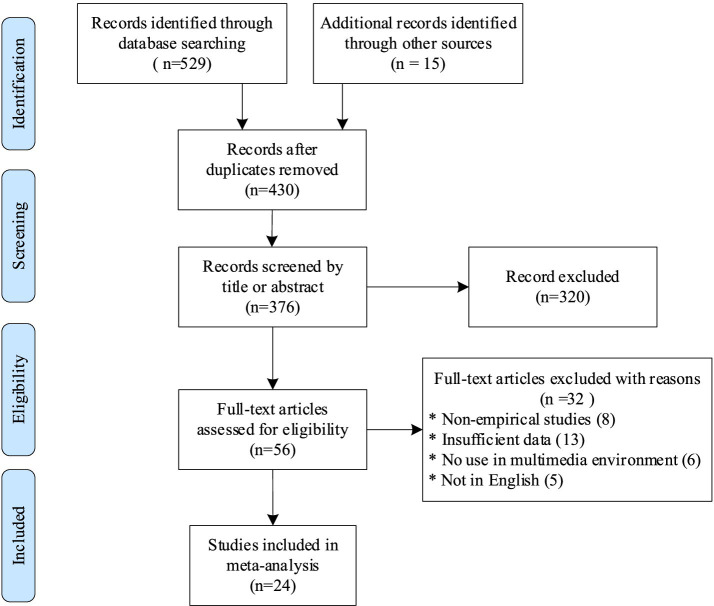
Flow diagram of the study included.

The screening process was divided into four stages: (1) Identification: Duplicate records were retrieved and removed. (2) Screening: Titles and abstracts were screened against the inclusion criteria (3) Eligibility: Records that passed the screening were examined in their entirety (including those that could not be screened by title and abstract only). (4) Inclusion: Studies that met the inclusion criteria were included in the meta-analysis. As shown in Figure 1, 24 records ultimately met the inclusion criteria for this meta-analysis.

### 3.3 Coding procedures

Coding was done by the first author and reviewed by all authors. The following literature characteristics were extracted from each study: author, year of publication, type of pedagogical agent, role, voice, expression, movement, subject, intervention time, and learning pace. The coding details for the moderator variables is shown in Table 1 and the coding results are shown in Table 2.

**Table 1:** Coding classification.

Moderating variable	Coding details
PA Appearance	Cartoon, Human-like, Real Human
PA Role	Explanation, Guidance
PA Voice	Synthesized, Real Voice
PA Expression	Unchanged, Changing
PA Body Movement	With Movement, No Movement
Subject Domain	Natural Sciences, Social Sciences, Humanities, Engineering and Technology
Form of Media Mix	Picture/Text, Picture + Text
Intervention Time	<10 min, 10–30 min, >30 min
Learning Pace	Self-paced, System-paced

**Table 2:** Coding table for included studies.

Study	Appearance	Role	Expression	Voice	Movement	Subject Domain	Form of Media Mix	Intervention Time (min)	Learning Pace	Hedge‘s g
Van Mulken et al. (1998)	C	G	U	S	N	N	P + T	>30	SE	0.61
Choi and Clark (2006)	C	E	C	R	M	H	P + T	10 ~ 20	SE	0.13
Park (2015)	C	E	U	R	N	S	P/T	10 ~ 20	SE	−0.31
Yung and Paas (2015)	C	E	U	R	N	N	P/T	>30	SE	−0.01
Dinçer and Doğanay (2017)	C	G	U	R	N	E	P/T	>30	SE	−0.45
Lin (2020)	R	E	U	S	N	N	P + T	>30	SY	0.03
Johnson et al. (2015)	C	G	U	R	M	N	P + T	>30	SE	−0.16
De Melo et al. (2020)	H	G	C	S	M	E	P + T	>30	SE	−1.07
Huang and Mayer (2016)	H	E	U	R	N	E	P + T	>30	SE	0.1
Tan et al. (2020)	H	G	U	R	N	S	P/T	10 ~ 20	SY	0.1
Ba et al. (2021)	H	G	C	S	N	E	P/T	>30	SE	−0.02
Wang (2022) Exp. 1	R	E	C	R	N	E	P + T	>30	SY	−0.33
Wang (2022) Exp. 2	R	E	U	R	N	E	P + T	>30	SY	0.57
Colliot and Jamet (2018)	R	E	U	R	N	H	P/T	<10	SE	−0.33
Sondermann and Merkt (2023a)	R	E	C	R	N	N	P/T	<10	SY	−0.23
Sondermann and Merkt (2023b) Exp. 1	R	E	C	R	N	H	P/T	>30	SY	−0.06
Sondermann and Merkt (2023b) Exp. 2	R	E	C	R	N	H	P/T	>30	SY	0
Wang et al. (2020a) Exp. 1	R	E	C	R	M	N	P + T	<10	SY	0.26
Wang et al. (2020a) Exp. 2	R	E	C	R	M	N	P + T	<10	SY	0.22
Wang et al. (2020b)	R	E	C	R	M	N	P + T	<10	SY	0.22
Anggraini et al. (2020)	R	E	C	R	M	N	P/T	10 ~ 20	SY	−0.07
Atkinson (2002) Exp. 1	C	G	U	-	N	N	P/T	10 ~ 20	SE	0.18
Atkinson (2002) Exp. 2	C	G	U	S	N	N	P/T	10 ~ 20	SE	−0.81
Atkinson (2002) Exp. 3	C	G	U	S	N	N	P/T	10 ~ 20	SE	−0.43
Frechette and Moreno (2010) Exp. 1	H	E	U	S	N	N	P + T	10 ~ 20	SY	0.45
Frechette and Moreno (2010) Exp. 2	H	E	C	S	N	N	P + T	10 ~ 20	SY	0.43
Frechette and Moreno (2010) Exp. 3	H	E	U	S	M	N	P + T	10 ~ 20	SY	0.2
Frechette and Moreno (2010) Exp. 4	H	E	C	S	M	N	P + T	10 ~ 20	SY	0.37
Lusk and Atkinson (2007) Exp. 1	C	G	C	S	M	N	P/T	>30	SY	0.06
Lusk and Atkinson (2007) Exp. 2	C	G	U	S	N	N	P/T	>30	SY	0.46
Lusk and Atkinson (2007) Exp. 3	C	G	C	S	M	N	P/T	>30	SE	0.12
Lusk and Atkinson (2007) Exp. 4	C	G	U	S	N	N	P/T	>30	SE	−0.58
Moreno et al. (2001) Exp. 1	C	E	U	S	M	N	P + T	10 ~ 20	SE	0.18
Moreno et al. (2001) Exp. 2	C	E	U	S	M	N	P + T	>30	SE	0.35
Wang et al. (2018) Exp. 1	C	E	U	R	M	E	P/T	<10	SY	0.09
Wang et al. (2018) Exp. 2	C	E	U	R	M	E	P/T	<10	SY	−0.34
Li et al. (2016)	C	E	U	R	M	E	P/T	<10	SY	−0.22

Appearance: C, Cartoon; H, Human-like; R, Real Human; Role: E, Explanation; G, Guidance; Voice: S, Synthesized; R, Real Voice; Expression: U, Unchanged; C, Changing; Body Movement: M, With Movement; N, No Movement; Subject Domain: N, Natural Sciences; S, Social Sciences; H, Humanities; E, Engineering and Technology; Form of Media Mix: P + T, Picture + Text; P/T, Picture / Text; Learning Pace: SE, Self-paced; SP, System-paced.

### 3.4 Statistical analysis

#### 3.4.1 Calculation of effect sizes and weights

In this study, we used the Integrated Comprehensive Meta Analysis V 3.0 (Brüggemann and Rajguru, 2022), which is specifically designed for conducting meta-analyses. The effect size chosen for this study was Hedge’s g, which is a standardized effect size based on the difference in means that takes into account differences in sample sizes and adjusts for them, and is therefore effective in reducing the effect of variance across studies, making the results of the analyses more robust. Absolute values of Hedge’s g ranging from 0 to 0.2 are considered small effect, between 0.2 and 0.8 for a medium effect, and between 0.8 and 1 for a large effect (Cohen, 1992).


$$\text{Hedge}\prime s\;g=\frac{M_{1}+M_{2}}{S_{\text{pooled}}}$$

Where $M_{1}$ and $M_{2}$ are the means of the two groups. $S_{\text{pooled}}$ represents the pooled standard deviation, calculated as:


$$s=\sqrt{\frac{(n_{1}-1)\cdot {{\mathrm{SD}}_{1}}^{2}+(n_{2}-1)\cdot {{\mathrm{SD}}_{2}}^{2}}{n_{1}+n_{2}-2}}$$

In this formula, $n_{1}$ and $n_{2}$ denote the sample sizes of the two groups, ${\mathrm{SD}}_{1}$ and ${\mathrm{SD}}_{2}$ are the standard deviations of each group.

#### 3.4.2 Heterogeneity analysis

Heterogeneity was evaluated using the I^2^ statistic and the chi-square test (Cochran’s Q statistic), which quantify the percentage variation in effect sizes. Following the guidelines by Higgins et al. (2003), an I^2^ value of 75% or more indicates high heterogeneity, 50% indicates moderate heterogeneity, and 25% indicates low heterogeneity. A threshold of 50% heterogeneity was applied to determine the use of either a fixed-effects model (<50%) or a random-effects model (>50%).

#### 3.4.3 Publication bias analysis

Publication bias is the tendency for studies with certain statistically significant or favorable results to be published more frequently, while invalid or less favorable results may be overlooked or ignored (Vevea and Woods, 2005). This phenomenon can lead to biased representation of the research field and potentially exaggerate the apparent effect size in meta-analyses. Funnel plots are an important visual tool for detecting publication bias because a symmetrical funnel shape indicates an unbiased study distribution, whereas an asymmetrical funnel shape may indicate potential bias. Specifically, smaller studies with different effect sizes should be distributed around the overall effect in a balanced manner. In addition, we used quantitative tests, including the Egger linear regression test and the Begg rank correlation test, to statistically assess publication bias. The Egger test assesses the asymmetry of the funnel plot through linear regression, whereas the Begg test uses the rank correlation to measure the relationship between effect estimates and their variance.

## 4 Results

### 4.1 Overall heterogeneity analysis

The results of the heterogeneity test showed a *Q*-value of 69.218, *p* = 0.001, and *I^2^* = 47.99% < 50%, indicating that the results were significantly heterogeneous and that the heterogeneity was at a moderately low level. Therefore, the fixed-effects model was used in this study.

### 4.2 Publication bias test

**Figure 2:**
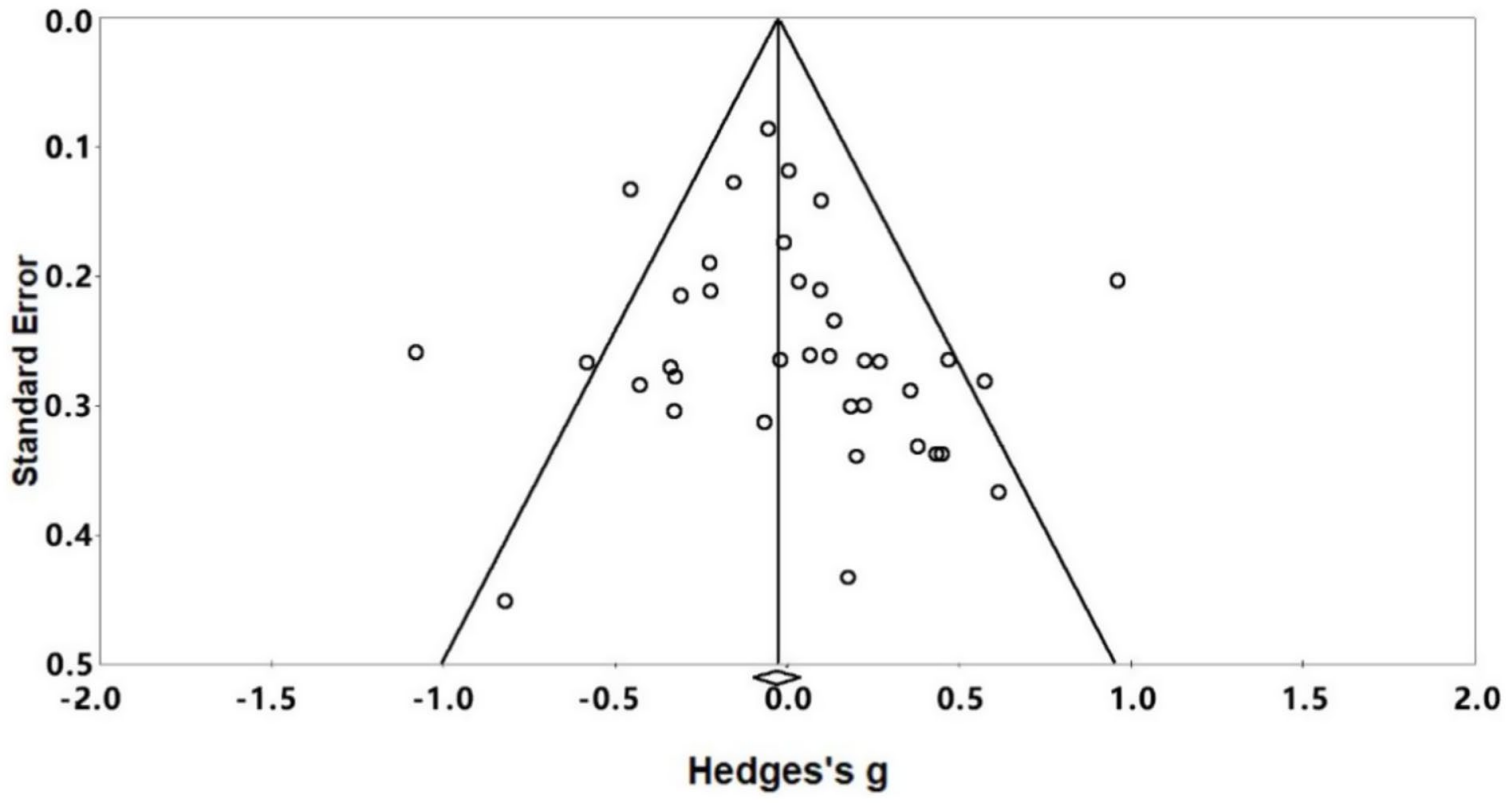
Publication bias test funnel plot.

As shown in the funnel plot of publication bias in Figure 2, most of the effect sizes were located in the upper part of the ‘inverted funnel’ and were more evenly distributed on both sides of the centre line, while the results of Egger’s linear regression test were *p* = 0.300 > 0.05, and the results of the Begg’s rank correlation test were *Z* = 1.54 < 1.96, *p* = 0.123 > 0.05, indicating that there was a publication bias in this study. 0.05, indicating that there is a low likelihood of publication bias in this study.

### 4.3 Main effect

In this study, the fixed-effects model was used to investigate the effect of pedagogical agent on cognitive load, and the effect size of the 37 studies combined was *g* = −0.053, with a 95% confidence interval of [−0.120, −0.014], *p* = 0.046. Meanwhile, ‘leave-one-out’ analyses revealed that no matter which study was removed individually, it did not have a significant effect on the overall effect size, which was stable between −0.062 and −0.024. Since the possibility of publication bias and small sample effects has been ruled out, this suggests that pedagogical agents would only slightly reduce cognitive load overall. Combined with the high degree of between-study heterogeneity derived from heterogeneity analyses (*Q* = 69.218, *p* < 0.001), it is reasonable to assume that the effect of pedagogical agent on cognitive load is largely influenced by potential moderating variables, which would need to be identified through moderated effects analyses. Forest plots of effect sizes and confidence intervals for all included studies are shown in Supplementary Figure 1.

### 4.4 Analysis of moderating variables

The analysis results for all moderator variable analysis is provided, allowing us to observe the results more easily, as shown in Table 3.

**Table 3:** Moderator analysis results.

Moderator	*k*	*r*	95% CI	*Q*	*Z*	*p*
PA appearance	37					0.199
Cartoon	18	−0.122	[−0.224, 0.020]	3.233	−2.337	0.019*
Human-like	8	0.028	[−0.135, 0.191]		0.135	0.736
Real human	11	−0.014	[−0.118, 0.091]		−0.118	0.795
PA role	37					0.011*
Explanation	24	0.011	[−0.073, 0.095]	6.400	0.205	0.838
Guidance	13	−0.174	[−0.289, −0.059]		−2.961	0.003**
PA voice	36					0.389
Synthesized	16	−0.001	[−0.140, 0.139]	0.742	−0.007	0.995
Real voice	20	−0.070	[−0.147, 0.006]		−1.808	0.071
PA expression	37					0.600
Unchanged	22	−0.069	[−0.161, 0.022]	0.275	−1.494	0.135
Changing	15	−0.034	[−0.132, 0.065]		−0.671	0.502
PA body movement	37					0.632
With movement	21	−0.064	[−0.145, 0.017]	0.229	−1.340	0.121
No movement	16	−0.029	[−0.147, 0.088]		−0.533	0.626
Subject domain	37					0.042*
Natural sciences	22	0.028	[−0.077, 0.133]	8.202	0.518	0.604
Social sciences	4	−0.036	[−0.554, 0.580]		−0.554	0.580
Humanities	2	−0.026	[−0.255, 0.204]		−0.220	0.826
Engineering & technology	9	−0.223	[−0.362, −0.085]		−3.160	0.002*
Form of media mix	37					0.023*
Picture/Text	20	−0.107	[−0.188, −0.025]	5.140	−2.569	0.010*
Picture + Text	17	0.058	[−0.059, 0.175]		0.974	0.330
Intervention duration	37					0.372
<10 min	8	−0.058	[−0.225, 0.108]	1.978	−0.687	0.492
10–30 min	14	−0.003	[−0.103, 0.098]		0.051	0.959
>30 min	15	−0.017	[−0.214, −0.001]		−1.978	0.048*
Learning pace	37					0.002**
Self-paced	20	0.032	[−0.054, 0.119]	9.332	0.735	0.462
System-paced	14	−0.180	[−0.285, −0.175]		−3.347	0.001**

* indicates *p* < 0.05, and ** indicates *p* < 0.01. Bold font indicates that the data is statistically significant.

In the present study, the samples were divided into three groups according to the pedagogical agent image: cartoon, anthropomorphic, and real person. The effect between groups *Q* = 3.233, *p* = 0.199 > 0.05, the difference between groups is not statistically significant, in which the effect of the pedagogical agent of the cartoon image on cognitive load reaches a significant level *g* = −0.122, *p* < 0.05, which indicates that it has a smaller degree of reduction of the cognitive load, anthropomorphic image effect size *g* = 0.028, *p* > 0.05, real person effect size *g* = − 0.014, *p* > 0.05, have no significant effect on students’ cognitive load.

The samples were divided into lecture combination guidance groups according to the role of pedagogical agent, and the analysis yielded the effect between groups *Q* = 6.400, *p* = 0.011 < 0.05, indicating that the effect of pedagogical agents with different roles on the cognitive load of the students has a significant difference in the impact of the effect of the students. Among them, the effect of pedagogical agent playing the role of guiding on cognitive load reaches a significant level *g* = −0.174, *p* < 0.05, indicating that it has a smaller degree of reduction of cognitive load, while the effect size of pedagogical agent playing the role of explaining *g* = 0.011, *p* > 0.05, indicating that it cannot significantly reduce cognitive load.

The samples were divided into two groups based on pedagogical agent voices: real human voice-over and machine synthesis, and one experiment that did not use voice-over was excluded (Atkinson, 2002). The effect between the two groups *Q* = 0.742, *p* = 0.389 > 0.05, and the difference between the groups was not statistically significant. The effect size of the pedagogical agent group that used live human voice overdubbing *g* = −0.070, *p* = 0.071, which was borderline significant, and the effect size of the pedagogical agent group that used machine synthesised voices *g* = −0.001, *p* = 0.995 > 0.05, which was not significant.

The samples were divided into two groups with and without changes based on pedagogical agent expressions. The effect between groups *Q* = 0.275, *p* = 0.600 > 0.05, and the difference between groups was not significant. The effect size of the pedagogical agent group with no change *g* = −0.069, *p* = 0.135, and the effect size of the pedagogical agent group with expression change *g* = −0.034, *p* = 0.502, which are not significant.

The sample was divided into two groups with and without movements based on pedagogical agent physical movements. The effect between groups *Q* = 0.632, *p* = 0.389 > 0.05, and the difference between groups was not significant. The effect size of the pedagogical agent group without movement *g* = −0.064, *p* = 0.121, and the effect size of the pedagogical agent group with movement *g* = −0.029, *p* = 0.626, neither of which is significant.

The sample was divided into four groups according to subject domains: natural disciplines, humanities, social disciplines, and engineering and technology. The effect between groups *Q* = 8.202, *p* = 0.042 < 0.05, the difference between groups is significant. The effect size of engineering technology group *g* = −0.223, *p* = 0.002, indicating that it has a moderate reduction of cognitive load, while the effect size of the other three subject domains is close to 0, and none of them is significant.

The samples were divided into two groups according to the media combination format: ‘picture / text’ and ‘picture + text’. If both pictures and text were presented in the same frame, it was considered the latter, and if only one of pictures (with appropriate legends and arrows) or text was presented, it was considered the latter. The results showed a significant between-group effect *Q* = 5.140, *p* = 0.023 < 0.05. Among them, the effect size of the picture/text group *g* = −0.107, *p* < 0.05, indicating that it has a moderate reduction of cognitive load, and the effect size of the picture + text group *g* = 0.058, *p* > 0.05, indicating that it cannot reduce the cognitive load.

The samples were divided into < 10 min, 10 ~ 30 min and >30 min groups according to the intervention time, and the effect between groups *Q* = 1.978, *p* = 0.372 > 0.05, and the difference between groups was not statistically significant. The effect size *g* = −0.017 (*p* = 0.048) for the >30 min group indicates that multimedia learning at intervention times longer than 30 min has a lesser degree of reduction in cognitive load, while the effect sizes *g* = −0.058 (*p* > 0.05) for the <10 min group and *g* = −0.003 (*p* > 0.05) for the 10–30 min group are not significant.

The samples were divided into two groups, systematic pacing and self-paced pacing, according to the learning pace. The effect between groups *Q* = 9.332, *p* = 0.002 < 0.05, the difference between groups is significant. The multimedia learning effect size of self-paced *g* = −0.180, *p* < 0.05, which indicates that it has a smaller degree of reduction of cognitive load, and the effect size of the systematic pacing group *g* = 0.032, *p* > 0.05, which is not significant for the reduction of cognitive load.

## 5 Discussion

### 5.1 The main effect of pedagogical agents on cognitive load

The results of the main effects of this meta-analysis showed g = −0.053, indicating that the presence of a pedagogical agent had a small to negligible effect on reducing cognitive load. In fact, 23 out of a total of 37 studies from 24 papers included in the literature showed no significant effect on learners’ cognitive load with the intervention of a pedagogical agent. The reason for this result is most likely that pedagogical agents as a pedagogical tool that provides social cues and gives learners emotional support itself facilitates learners’ cognitive processing, but as it also acts as a visual element in addition to the main learning content in multimedia learning, competing for the learners’ cognitive resources, it results in the positive and negative effects cancelling each other out that makes the effect size at a very low level. Although the effect size is not significant overall, combined with the heterogeneity of the studies included in the meta-analysis, it side-steps the fact that the effect of pedagogical agents on cognitive load is largely influenced by certain moderating variables that must be explored in depth.

### 5.2 Moderating effects of pedagogical agents affecting cognitive load

Six of the nine moderating variables analyzed in this study were significant: pedagogical agent appearance, pedagogical agent role, Subject Domain, form of media mix, intervention duration, and learning pace. By controlling for these moderating variables in a multimedia environment, the instructional agent is able to reduce the cognitive load on the learner more consistently.

#### 5.2.1 The moderating role of pedagogical agent appearance

In terms of the image of pedagogical agents, there are some differences in the effects of different types of pedagogical agents on students’ cognitive load. Pedagogical agents with cartoon images will reduce students’ cognitive load. This may be due to the fact that cartoon images, which are usually characterized by simplification and exaggeration, are more likely to attract students’ interest and attention, thus reducing the cognitive effort they need to make sense of and process the information (Choi and Clark, 2006). The pedagogical agents of anthropomorphic (*g* = 0.028) and real-life (*g* = −0.014) images did not seem to have a significant effect on students’ cognitive load. This may be due to the fact that real-life and anthropomorphic images may be more complex and have more details, and the specific effects may be affected by a variety of factors, such as detailed facial expressions, movements, and mouth patterns. In addition, the agency of certain anthropomorphic and real-life images may stress some students and make them feel the social pressures or standards associated with them, making the overall effect of reducing cognitive load less pronounced.

#### 5.2.2 The moderating role of pedagogical agent roles

In terms of agency, the pedagogical agent of the lecturer did not significantly reduce the cognitive load of students, but even slightly increased it (*g* = 0.011). This may be attributed to the lack of interaction and learner autonomy in lecture-style presentations, which leads to passive reception of information and cognitive disengagement, with learners continuously exposed to language input. Furthermore, from the perspective of the redundancy principle (Mayer, 2002), if pedagogical agents simply repeat information already presented on the screen or in the narration, this may increase processing demands without adding meaningful value. In contrast, pedagogical agents that play a guiding role in the learning process have been found to significantly reduce learners’ cognitive load (*g* = −0.174). This can be explained by social agency theory (Mayer and DaPra, 2012), which argues that agents that provide personalized guidance, prompts, or scaffolding create a sense of social partnership and interaction. This sense of social presence enhances learners’ motivation and attention, helping to manage internal cognitive load and reduce unnecessary mental effort. Additionally, guiding agents typically apply segmentation and signaling principles, breaking down complex content into manageable parts and directing attention toward key elements—both of which support more efficient information processing and reduce cognitive demands (Mayer, 2002).

#### 5.2.3 The moderating role of subject domain

In terms of subject domain, the effectiveness of pedagogical agents on cognitive load varies, likely due to differences in cognitive task demands. In natural science subjects, agents were associated with a slight, non-significant increase in cognitive load (*g* = 0.028). According to cognitive load theory, these subjects often involve high intrinsic load from tasks such as conceptual abstraction and experimental reasoning (Sweller, 2011). When baseline demands are high, additional affective or redundant cues from agents may introduce extraneous load that interferes with learning. This aligns with Wang et al. (2020a), who found agents can hinder performance in complex tasks when not well-aligned with learning goals. In contrast, the humanities (*g* = −0.036) and social sciences (*g* = −0.026) showed small, non-significant reductions in cognitive load. While these disciplines emphasize deep reasoning and knowledge construction, the emotional and social cues offered by pedagogical agents may not significantly support cognitive efficiency unless they directly enhance information processing. According to the Cognitive-Affective Theory of Learning with Media, emotional support aids learning only when it facilitates meaningful cognitive engagement (Moreno, 2006). A larger reduction in cognitive load was observed in engineering and technology subjects (*g* = −0.223). These disciplines typically follow a learning-by-doing model focused on procedural tasks and practical problem-solving. Here, pedagogical agents can act as cognitive guides, providing step-by-step support aligned with the segmenting and modality principles. Learners also benefit from concrete instructional scaffolding (Paivio, 1991), as in Dinçer and Doğanay (2017), where guided agent support improved learners’ software training outcomes by reducing anxiety and enhancing perceived competence (Plass and Kalyuga, 2019).

#### 5.2.4 The moderating role of form of media mix

The use of pedagogical agents significantly reduces students’ cognitive load when the medium of the learning content is one of pictures or text (*g* = −0.107). The possible explanation is that when the learning material is visually relatively simple and less informative, the presentation of pedagogical agents can provide students with additional supporting information, explanations, or guidance to help them better understand and assimilate the knowledge, thus reducing the cognitive load. On the contrary, the use of pedagogical agents may lead to an increase in students’ cognitive load when the learning material contains both pictures and text (*g* = 0.058). Consistent with the findings of Wang et al. (2020a), pedagogical agents increased students’ cognitive load in graphic instructional videos on complex topics designed for statistical knowledge, while pedagogical agents reduced students’ cognitive load in videos on simple topics. This may be due to the fact that the learning material already occupies enough visual processing channels for students, and the presentation of pedagogical agents may have increased their cognitive load by increasing the processing burden of visual information or by drawing their attention to unnecessary information. Dinçer and Doğanay (2017) also pointed out that the improvement of cognitive load by pedagogical agents is effective only if the principles of good multimedia screen design are followed. Therefore, when designing pedagogical videos, it is necessary to consider whether to use pedagogical agents and how to present these images according to the complexity and the amount of information in the learning material in order to maximise students’ learning outcomes.

#### 5.2.5 The moderating role of intervention duration

The effect of learning duration on the cognitive load of pedagogical agents on students may be related to changes in students’ attention and their adaptation to the emotional and social cues provided by pedagogical agents. The use of a pedagogical agent was able to reduce cognitive load when the duration of study was <10 min (*g* = −0.058). This is due to the fact that students’ attention can be at a high level of concentration for a short period of time, as the novelty effect induces a high level of engagement and allows for greater comprehension and assimilation of the content (Clark, 1983; Guo et al., 2014). At this time cognitive resources are also able to withstand the additional emotional and social cues presented by the pedagogical agent, helping students to understand the learning content better and thus reducing cognitive load. The decrease in the reduction of cognitive load (*g* = −0.003) for learning durations between 10 and 30 min may be due to the fact that as the learning time increases, students’ attention begins to decline, and their ability to comprehend and assimilate the content gradually decreases. The use of pedagogical agents was again able to reduce the cognitive load slightly (*g* = −0.017) for learning durations >30 min, most likely because with longer learning durations, students may have gradually become familiar with and established certain social relationships with the pedagogical agent, and therefore were able to quickly comprehend the emotional and social cues provided by the pedagogical agent, and the allocation of cognitive resources is reduced, at which point they may even focus more on the learning content itself and the perceived overall cognitive load is naturally reduced. Grivokostopoulou et al. (2020) used embodied pedagogical agents in a virtual 3D environment and found that students who studied longer had a better understanding of the content and topics of the course. Dempsey et al. (2004) also found that learners preferred pedagogical agents that were already more familiar to their senses to help them learn.

#### 5.2.6 The moderating role of learning pace

When learning at a self-paced pace, the effect size of using pedagogical agents was negative (*g* = −0.180), with pedagogical agents reducing students’ cognitive load to a lesser extent. In an autonomous learning environment, learners have greater control over the speed at which they process information, which can alleviate time pressure and enable more efficient allocation of cognitive resources. In such cases, instructional agents can further reduce cognitive load by guiding attention, simplifying complex materials, or providing emotional support—functions consistent with social agency theory, which posits that social cues such as facial expressions and voice can promote deeper engagement and understanding (Mayer and DaPra, 2012). In contrast, in system-paced learning where time constraints are externally imposed, learners may have less flexibility to regulate their processing, which could lead to increased additional load. In such cases, if instructional agents introduce unnecessary or poorly timed information, thereby violating principles such as modality and coherence that emphasize minimizing redundant or distracting content, they may become less effective or even counterproductive (Mayer, 2002). This may help explain the smaller and slightly positive effect observed under system-paced conditions (*g* = 0.032).

## 6 Limitations and future directions

For the research nature of the meta-analysis, this study was only able to include variables that were explicitly reported in the original literature and had extractable statistical indicators. Unfortunately, most of the included studies did not systematically report or analyze subgroups for characteristics such as participants’ age, background, or level of knowledge, limiting our possibilities to conduct further analyses of these internal variables. Future studies should report participant characteristics more systematically so that subsequent meta-analyses can examine the potential moderating effects of intra-learner factors such as age, prior knowledge, and educational background.

In this study, the coding of pedagogical agents’ expressions and body movements could only be differentiated between the presence or absence of expressions and the presence or absence of body movements because the included studies did not meticulously describe the agents’ expressions and body movements, and in fact the specific presentation of expressions and body movements as a kind of complex social cues may also have an impact on the cognitive load of the students. At the same time, due to the different levels and generations of research with different levels of maturity of technological tools, if the agent’s voice, expression, or body movement is not presented prominently enough or realistically enough, it may not be able to elicit an affective response from the students, and thus may result in the failure to detect significant moderating effects of all three on cognitive load, and future research on pedagogical agents should pay attention to these detail-oriented social cues.

## 7 Conclusion

This study conducted a meta-analysis on how the presence of pedagogical agents in multimedia learning affects learners’ cognitive load. The main effect analysis showed that the pedagogical agent had a small or even negligible effect on reducing cognitive load. The moderator variable analysis showed that cartoon pedagogical agents had a significant effect on reducing cognitive load, guided pedagogical agents could significantly reduce cognitive load, using pedagogical agents in engineering and technology could significantly reduce cognitive load, and only presenting pictures or text could also help reduce cognitive load. Instructional surrogates with interventions lasting less than 10 min or more than 30 min slightly reduced cognitive load. Finally, the use of pedagogical agents in self-paced learning could reduce cognitive load. These findings provide practical implications for instructional designers that in order to reduce the cognitive load on learners, the following variable controls can be used in the design of multimedia learning environments for different contexts:(1) use cartoon-style, guided agents; (2) use pedagogical agents in engineering or procedural tasks; (3) try to make sure that pictures and text do not appear on the screen at the same time when using an agent (4) the use of an agents for interventions should ideally be longer than 30 min (5) incorporating teaching agents in self-paced learning environments.

## Data Availability

The original contributions presented in the study are included in the article/Supplementary material, further inquiries can be directed to the corresponding author.
