# Supplementary material for: The facts about the effects of pedagogical agents on learners’ cognitive load: a meta-analysis based on 24 studies
Source: Front Psychol. 2025 Jul 24;16:1635465. doi: 10.3389/fpsyg.2025.1635465 (PMC12328452; doi:10.3389/fpsyg.2025.1635465)
Supplement: Supplementary file 1 [file Supplementary_file_1.docx]

Supplementary Material


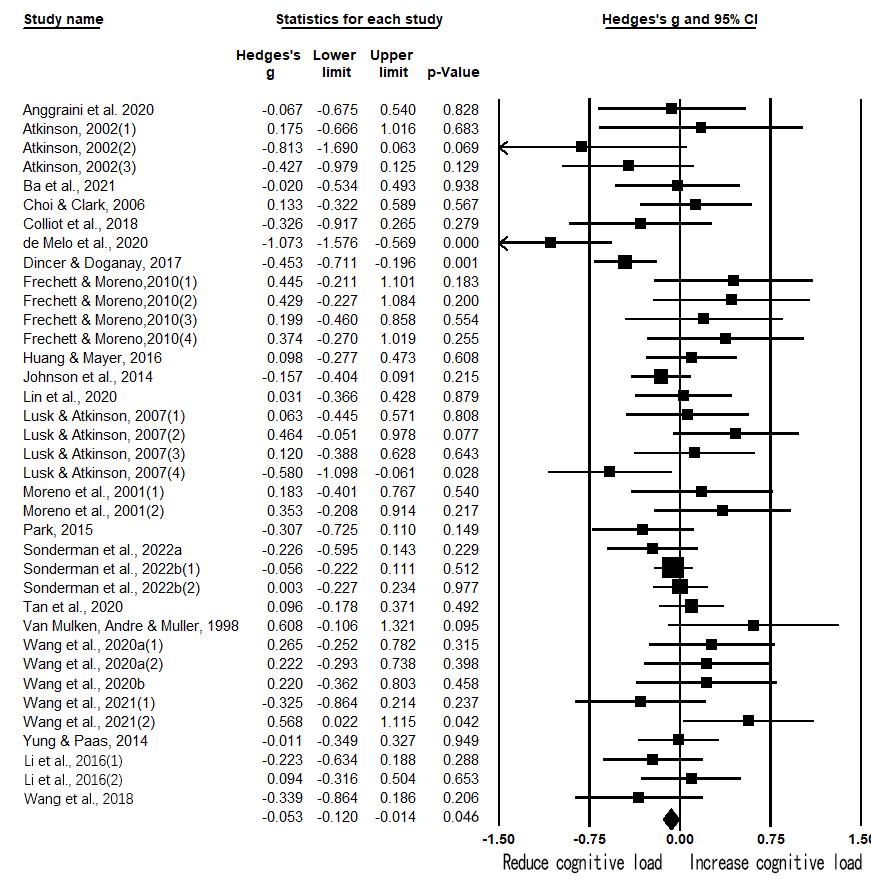


**Supplementary Figure 1.** Forest Plot of All Included Studies (fixed effects model)
